# Asymmetric interactions and feast–famine cycles drive chaos in microbial populations

**DOI:** 10.21203/rs.3.rs-8021262/v1

**Published:** 2025-11-18

**Authors:** Megan Behringer, William McLaughlin, Thomas Beardsley, Natalia Komarova

**Affiliations:** Vanderbilt University; Vanderbilt University; University of California - Irvine; University of California San Diego

## Abstract

Predicting the dynamics of ecological systems is a central challenge in biology. One factor that could contribute to limited predictability is the presence of ecological chaos. Theory has long suggested that even simple interactions could produce chaos, yet empirical demonstrations in living systems remain scarce. Here we show that chaos emerges naturally in a minimal microbial community exposed to feast–famine cycles. In a two-strain system of *Escherichia coli*, increasing the duration before replenishing resources reveals transitions from exclusion, to stable coexistence, to chaos, driven by asymmetric ecological interactions and periodic environmental forcing. A dynamical systems framework (under consumer-resource or much simpler generalized Lotka-Volterra models) both predicts and recapitulates these dynamics, confirming chaos under tractable laboratory conditions. Because feast–famine cycles and asymmetric **interactions** are widespread in nature, our findings provide a model for uncovering the ecological and evolutionary significance of chaos and reveal fundamental limits to predictability in living systems.

Microbes coexist in diverse and dynamic ecologies in virtually every habitat on Earth. This ecological diversity is not limited to the species level – heterogeneity is common within natural populations^3^, and intraspecific diversity can form through the formation of specialized subpopulations, or ecotypes, that are metabolically or spatially differentiated^4–7^. Understanding the conditions that influence or maintain this diversity has been of great interest for microbial ecologists^8^. Although mechanisms such as cross-feeding and niche partitioning explain coexistence in predictable settings^7,9–12^, longitudinal predictions about population dynamics remain a challenge. One barrier to predictability is the high degree of variation in resource availability and stress in natural microbial habitats, which can destabilize communities and shift outcomes between exclusion and coexistence^13^.

A potential explanation for these unpredictable dynamics is ecological chaos. Defined as deterministic behaviour characterized by aperiodic oscillations and sensitivity to initial conditions, chaos was first identified in ecological models more than 50 years ago^1^. Theory predicts chaos should be widespread in natural ecosystems^2^, but empirical demonstrations remain rare^14–17^ and have never shown a chaotic transition in bacterial population dynamics. Synthetic microbial ecology has elucidated principles of community assembly and coexistence^9,18–21^, but standard methods, including chemostats or rapid passaging, typically impose constant conditions, suppressing the variability inherent to natural habitats. As a result, we lack simple and tractable models to explore how microbial populations transition between exclusion, stable co-existence, and chaotic dynamics.

Natural microbial habitats experience periodic environmental turnover that is highly heterogeneous in both frequency and intensity. Feast–famine cycles represent a ubiquitous form of environmental turnover that microbes endure, ranging from environments like the human gut, to intertidal and deep-sea communities, to soil habitats^22–24^. Previous work in experimental populations of *E. coli* has shown that long feast–famine intervals can promote disequilibrium coexistence in populations^7^, whereas short intervals foster more stable population structures^6^. These observations suggest that the periodicity of resource turnover itself may dictate whether ecotypes coexist stably, exclude one another, or engage in more complex behaviours.

To predict and explain such dynamics, ecologists often turn to quantitative frameworks. Generalized Lotka–Volterra and consumer–resource models have been instrumental in predicting community outcomes and identifying drivers of instability^25^. Theory identifies three key features capable of generating chaos: non-reciprocal (asymmetric) interactions^26–29^, discrete dynamics^1,25^, and periodic forcing^30,31^ (where a force is regularly introduced to a system, akin to feast and famine cycles). Although these features are ubiquitous in microbial ecosystems, the effects of these parameters have not been experimentally demonstrated and assessed using quantitative tools. Our existing systems^7^ prove too complicated to reduce model dimensionality enough to isolate these determinants of population dynamics, and a simplified, tractable microbial community is needed.

To address this gap, we engineered a minimal two-strain *Escherichia coli* cross-feeding community composed of a methionine auxotroph (obligate consumer) and an overproducer (obligate provider). By subjecting these populations to feast–famine cycles of different periodicities, we tracked ecotype frequencies over time and directly tested whether chaos can emerge from simple ecological interactions under fluctuating environments.

## Results

### Feast-famine cycles drive stable vs. unstable co-existence

**Figure 1:**
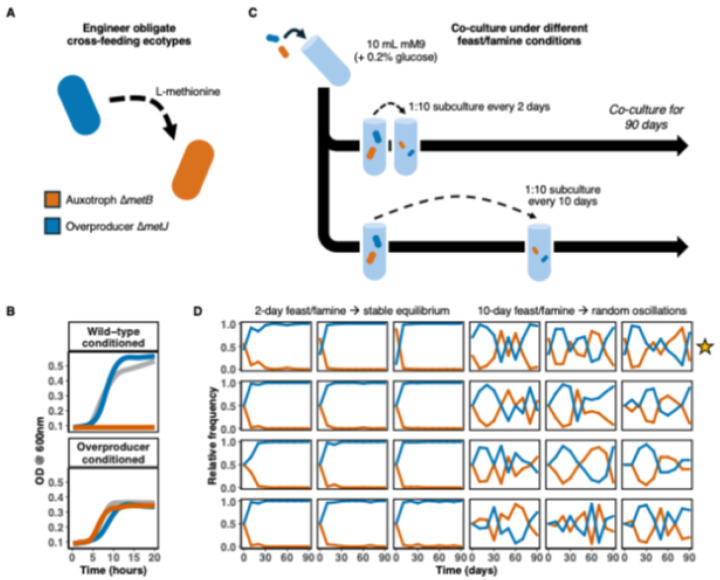
Lengthening feast and famine periodicity drives oscillations. (**A**) Schematic of engineered mutant strains of E. coli K-12 composed of a methionine overproducer Δ*metJ* (blue) and a methionine auxotroph Δ*metB* (orange). A bold dotted arrow indicates the obligate exchange of methionine from overproducer to auxotroph. (**B**) Growth curves (measured by optical density at a wavelength of 600 nm) for both mutant strains and wild-type in the conditioned media of the overproducer Δ*metJ* and wild-type. Gray shading indicates 95% confidence intervals. (**C**) Experimental design and sampling schedule for 90-days of culture in starved and un-starved conditions. See Methods for culture conditions. (**D**) Relative frequency of each ecotype quantified every 10 days for the duration of the experiment.

We engineered one-way methionine cross-feeding populations of *E. coli* K-12, consisting of an auxotroph (Δ*metB*, lacking the first gene in the methionine de novo biosynthesis pathway^32^) and an overproducer (Δ*metJ*, lacking the repressor of de novo methionine biosynthesis^33,34^; Fig. 1A). To verify that the auxotroph grows only in the presence of the overproducer, we cultured each mutant and wild-type strain in conditioned (spent) modified M9 minimal medium (mM9). Wild-type conditioned medium supports only the wild-type and overproducer, whereas overproducer conditioned medium supports all three strains, rescuing auxotrophic growth (Fig. 1B).

To assess the effect of feast and famine periodicity, we cultivated 24 replicate co-cultures in 10 mL of mM9 with 0.2% glucose, subjecting cultures to a 1:10 bottleneck into fresh media either every 2 or 10 days for 90 days (Fig. 1C). In the 2-day subculture condition, the ecotypes converge to a stable equilibrium where the overproducer comprises the majority of the overall cell density (~99% overproducer, ~1% auxotroph) (Fig. 1D, left). Alternatively, the 10-day subculture populations exhibit highly variable, oscillating ecotype frequencies (Fig. 1D, right). These results suggest that extended periods between subcultures contribute to driving complex, seemingly chaotic population dynamics.

### Carryover and growth strategies underlie oscillating frequencies in 10-day cultures

**Figure 2:**
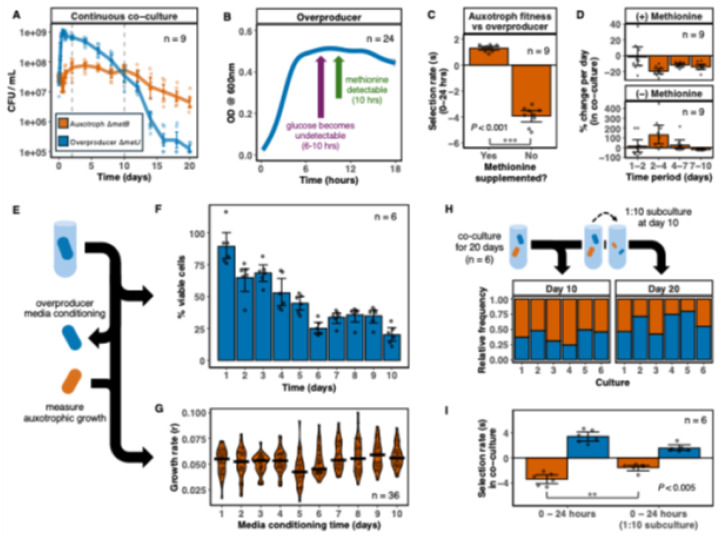
Auxotroph and overproducer strains display differentiated growth strategies that are sensitive to carryover effects. (**A**) 20 days of auxotroph (orange) and overproducer (blue) co-culture in mM9 + 0.2% glucose. Dashed lines indicate days 2 and 10. (**B**) Growth (measured by optical density at 600 nm wavelength) of the overproducer in unsupplemented mM9 + 0.2% glucose. Gray shading indicates 95% confidence intervals. Arrows indicate glucose undetectability (purple) and methionine detectability (green) (see fig. S3). (**C**) Auxotroph selection rate across an initial 24 hour period in overproducer co-culture (+/−) L-methionine. Lack of methionine yields significant decrease in auxotroph fitness in co-culture. (Welch’s two sample t-test, t = −20.713, P = 1.6e-9, df = 9.96) (**D**) Periodic growth rate (measured in percent CFU/mL change per day) of the auxotroph across 10 days in overproducer co-culture (+/−) L-methionine. (**E**) Overproducer viability and auxotrophic growth rate experimental design. (**F**) Viability of overproducer cells in monoculture across 10 days. (**G**) Growth rate of the auxotroph in pooled spent media (n = 6 per day) of the overproducer across 10 days (black bars indicate the median). (**H**) Strain relative frequencies at 10 (left) and 20 days (right) of co-culture (1:10 subculture at day 10). (**I**) Strain selection rates across an initial 24 hour period in co-culture (days 0 to 1) and 24 hours following a 1:10 subculture (days 10–11). Auxotrophic fitness was significantly increased (Welch’s two sample t-test, t = 3.8709, P = 0.004, df = 8.309) following the subculture. All error bars indicate 95% confidence intervals.

We next sought to understand the ecotypic interactions underlying these oscillations. Monitoring 20-day co-cultures revealed that the overproducer dominates in the first 48 hours (~90% of the population; Fig. 2A). However, obligate methionine overproduction incurs a fitness cost (**fig. S1**), and the overproducer density declines after day 1 (Fig. 2A, **fig. S2**). The auxotroph displays a delayed recovery in co-culture, growing between days 2–6 and maintaining its abundance through day 10 (comprising 57% of the population on average; Fig. 2A, **fig. S2**). This delay explains the divergent patterns observed across 90-day experiments: in 2-day intervals, the auxotroph is subcultured at relatively low abundance (2–18%), whereas in 10-day intervals, prolonged culture leads to a more varied range of auxotrophic frequencies (29–77%) before subculture.

We hypothesized that the delay in auxotrophic growth arises due to competition for carbon resources. Both strains compete for glucose, but the auxotroph cannot grow until the overproducer supplies methionine. Consistent with this, measurements of extracellular glucose and methionine in overproducer monocultures confirm that glucose is depleted by 6–10 hours and methionine only accumulates consistently immediately thereafter (Fig. 2B, **fig. S3**). Supplementing methionine eliminates the growth delay, and the auxotroph outcompetes the overproducer in the first 24 hours (Fig. 2C). These results confirm that delayed growth is driven by methionine availability rather than glucose access.

Interestingly, although the auxotroph performs poorly in the first 24 hours in unsupplemented media (Fig. 2D), it maintains its population density across 4–10 days, demonstrating a trade-off between initial fitness and long-term survival (Fig. 2D). Since *E. coli* cannot metabolize methionine as a primary carbon source^35^, the auxotroph likely exploits secondary carbon sources, including other metabolic byproducts or necromass released upon overproducer cell death. In order to compare the proportion of dead overproducer cells with growth capacity in the auxotroph, we cultured the overproducer in isolation for up to 10 days and quantified the ratio of viable cells each day. Viability assays indicate overproducer cultures remain >60% viable for the first 3 days, suggesting early auxotroph growth relies on metabolic byproducts, but as the culture viability drops below 50% at day 5, necromass becomes a great contributor to carbon resources (Fig. 2E–F). Median auxotroph growth rates across conditioning times show no discernible trend (Fig. 2G), indicating flexibility in utilizing available resources. Together, these observations reveal complementary growth strategies: the overproducer relies on initial glucose, while the auxotroph capitalizes on secondary carbon sources.

Carryover of methionine, byproducts, and necromass during subculture further shapes early competitive dynamics. In 20-day co-cultures with a 1:10 subculture at day 10, ecotype frequencies vary widely from day 10–20, with the auxotroph becoming rarer overall (Fig. 2H). The introduction of carryover products alters initial fitness asymmetrically, enhancing auxotroph growth while reducing the density of the overproducer (Fig. 2I). As such, the carryover in each subculture will uniquely affect the starting conditions of each period, in turn altering the final ecotype distributions, explaining the variable population dynamics observed under 10-day feast–famine cycles.

### Chaos emerges from simple consumer-resource modeling

**Figure 3:**
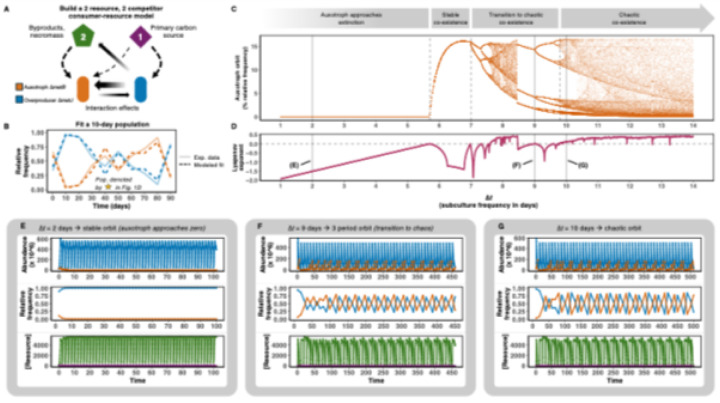
Simple consumer-resource modeling reveals transitions between exclusionary, stable, transition-to-chaotic, and chaotic ecotype dynamics. (**A**) Graphical representation of the consumer-resource model. Purple diamond represents primary limiting carbon (glucose), and green pentagon represents secondary carbon (byproducts and necromass) generated by the overproducer (blue), indicated by a skinny solid arrow. Dotted arrows directed towards each microbe indicate consumption, and the boldness of the arrow correlates with the consumption rate. Solid arrows between the microbes are scaled for the size of the effect each strain has on the other in the model. (**B**) Modeled data (dark dashed lines) fitted to empirical results from a 10-day population indicated in Fig. 1D (pale solid lines). (**C**) Bifurcation diagram demonstrating the orbit of the auxotrophic strain in the calibrated model generated in panel B. Each value of Δt (represents the frequency of a 1:10 subculture) was iterated 100 times. Solving the model from 1 through 14 ‘day’ subculture frequencies reveals transitions between auxotrophic exclusion, stable co-existence, chaotic transition, and chaotic co-existence (dotted gray lines indicate transitions between these different co-existence patterns). Solid gray lines indicate the *Δt* value of each example represented in panels E, F, and G. (**D**) Corresponding Lyapunov exponents aligned with the bifurcation diagram for each *Δt* value across a range of dilution frequencies. A Lyapunov exponent above 0 indicates a chaotic orbit, whereas below 0 indicates a stable or periodic orbit. (**E**) Dynamics of the model at a *Δt* = 2 days, which result in a stable orbit where the auxotroph relative frequency approaches zero. The top row displays the abundance of the two ecotypes, middle row displays the relative frequency measured at the end of each growth period, and bottom row indicates the resource concentration (arbitrary units). (**F**) The model under Δt = 9 days predicts a stable limit cycle with a 3-period orbit for the auxotroph relative frequency, a classic signature of transitioning into chaos. (**G**) The model under *Δt* = 10 days predicts chaos, where the auxotroph relative frequency displays aperiodic oscillations.

To generalize these population dynamics, we implemented a 2-species, 2-resource consumer-resource (CR) model with Monod growth dynamics (Fig. 3A; see Supplementary Information for additional details). In their classical formulation, CR models describing long-term uninterrupted co-cultures show convergence to a steady state, and no chaotic behaviour is observed. To recapitulate the setup of the microbial co-culture experiments, we incorporated serial dilutions into the model. To do so, we created a hybrid model where the growth between dilutions is described by ordinary differential equations (ODEs), and the dilutions are used to construct a discrete map whose dynamics mirror the experimental measurements of the co-cultures (Fig. 1D). This introduces two key features into the model: 1) discrete-time dynamics and 2) periodic forcing. Together with the asymmetric interactions between the overproducer and the auxotroph, these factors provide the ingredients for chaotic behaviour.

We parameterized the model by fitting it to co-culture time-series (Fig. 2A) and the 10-day dilution data. The resulting system, which reproduces the experimental population dynamics (Fig. 3B), was interrogated for chaotic behaviour, as the dilution interval (*Δt*) was varied (**Movie S1**). The bifurcation diagram of the auxotroph relative frequencies across dilution intervals (Fig. 3C–D) shows large regions of chaotic behaviour. Here, single or grouped clean lines indicate stable solutions or periodic stable limit cycles, whereas large patches of randomly distributed points signal chaotic dynamics. The bifurcation diagram reveals that at short dilution intervals, such as *Δt* = 2, the auxotroph goes extinct (Fig. 3E). Alternatively, at intermediate intervals, stable coexistence occurs, and at longer intervals, such as *Δt* = 10, the system undergoes period doubling and classical transition to chaos (Fig. 3F–G). The corresponding Lyapunov exponents confirm these transitions (exponent >0 indicates chaos, whereas <0 indicates stability or periodic oscillations) (Fig. 3D).

Remarkably, chaos is robust to model formulation. Much simpler generalized Lotka-Volterra equations with predator-prey-type interactions and periodic dilutions (which, like the experimental system, contain asymmetric interactions and periodic forcing) also yield a transition to chaotic dynamics (see Supplementary Information). These results demonstrate that chaos emerges in the presence of a small set of ingredients: periodic environmental forcing and asymmetric competition are sufficient to generate chaos in microbial communities.

## Discussion

Here, we demonstrate that ecological chaos can arise from just two interacting microbial ecotypes exposed to periodic resource fluctuations, resulting in the first tractable system to investigate how ecological perturbations drive chaotic population dynamics. Our culture experiments and metabolite assays show these patterns are largely driven by the timing of resource turnover and divergent ecotypic growth strategies that manifest during extended famine periods. Asymmetric feeding interactions change with every subculture, altering ecotype fitness and introducing variations in initial culture conditions that drive unique growth patterns each period. We identified the broad niches occupied by each strain, and by applying a simple consumer-resource model incorporating serial subculturing, we quantified chaos emerging between *Escherichia coli* ecotypes absent evolutionary processes.

Our results suggest chaos could be widespread in microbial habitats, as the stressors we recapitulated are common in nature. The effects of feast and famine are universal; timing and quality of nutrients in the human gut affect microbiome stability and composition^22,36–38^, drought or plant-microbe related nutrient influxes shape soil bacterial diversity^24,39,40^, and patchiness in marine nutrient availability drives booms and busts in microbial populations^23,41^. Microbial ecosystem stability and invasibility are sensitive to the rates and frequencies of fluctuations, the strength of interactions (e.g. cross-feeding), and competition for shared resources^42–45^. Given that asymmetric interactions like cross-feeding are widely distributed^46^, chaos may play a larger role in microbial species composition than previously appreciated. Nonetheless, while theory predicts that chaos is common^2^, extensive longitudinal monitoring is needed to confirm whether microbial populations in naturally variable environments frequently display chaotic dynamics.

Chaos can also act as a mechanism for co-existence. In conspecific communities with overlapping niches, competing ecotypes must either dominate a shared niche or differentiate sufficiently to co-exist. The dynamics between our engineered ecotypes mirror co-existence strategies observed in diverse habitats, such as the bee gut or mammalian rumen^47,48^, where rapid and slow growth specialists coexist. Importantly, our study suggests the existence of a ‘goldilocks zone’ of turnover frequency: intermediate feast–famine cycles promote chaotic co-existence, rapid subculturing favors independent producers, and our modelling predicts that slow turnover in lengthier feast and famine intervals will inevitably lead to an extinction event. Now, with a tractable experimental system, long-term chaotic co-existence presents an exciting new avenue for study. Effects of fluctuating or oscillatory population sizes, potentially selection-agnostic genetic sweeps, and eco-evolutionary feedbacks over long timescales can now be probed using our tools to investigate how chaos shapes the evolution of communities through time.

Over the last several decades, mathematicians have been fascinated by the possibility of chaotic dynamics in ecological systems. Versatile frameworks, like generalized Lotka–Volterra (gLV) and consumer-resource (CR) models, have enabled researchers to identify a variety of structural features that can give rise to chaotic behaviour^25^. It has been shown mathematically that in continuous systems, chaos emerges in communities with many components: purely competitive gLV systems require four or more species^49,50^, and standard CR models must have five or more species^51^ or three or more resources^52^ to show chaotic behaviour. Remarkably, our CR system achieves chaos with only two species and two resources, and the gLV model shows a chaotic transition with only two species. Here, chaos emerges after a series of bifurcations as the dilution interval increases, which changes the co-dynamics of the two species. This aligns with theoretical predictions that asymmetric interactions, discrete dynamics, and periodic forcing can foster chaos, as seen in models incorporating seasonal variation^53–55^.

Given the simplicity of our co-culture system and model, chaos may be an emergent feature of interdependent bacterial populations in a myriad of diverse habitats undergoing environmental turnover. Ecological communities are constantly interacting, changing, and evolving. By independently modifying the strength of asymmetric interactions and the frequency of environmental fluctuations, we identified two basic ingredients for ecological chaos. This system provides a platform to systematically explore the environmental bounds of chaotic behaviour in microbial populations.

## Materials and Methods

### Strain Construction

The genes of interest for construction of the cross-feeding strains were identified through the EcoCyc database^56^ by filtering for annotations indicating essentiality in minimal medium. The auxotroph *ΔmetB* strain was derived from PFM2, a prototrophic derivative of the *Escherichia coli* K-12 str. MG1655 reference strain^57^, provided by Dr. Patricia Foster (Indiana University). PFM2 served as the wild-type background for all experiments. The auxotrophic strain was generated via P1 phage transduction using deletion mutants from the Keio Collection^58,59^. To construct the methionine auxotroph, the *ΔmetB*726::kan allele was transduced into PFM2 from *E. coli* K-12 BW25113::*ΔmetB*. The methionine overproducer was created by excising the Δ*metJ*725::*kan* cassette from *E. coli* K-12 BW25113::*ΔmetJ*. The kanamycin resistance cassette was excised from the *ΔmetJ* strain using the FLP recombinase expressed from the helper plasmid pCP20, leaving behind a single FRT scar^60^. The *ΔmetB* strain retained its kanamycin resistance marker, enabling selective enumeration of auxotrophic cells in co-culture experiments when their numbers fell below the detection threshold of CFU counting on tetrazolium arabinose agar plates (TA agar). As a neutral genetic marker to differentiate strains on TA agar indicator plates, the *ΔmetJ* strain carries a Δ(*araD-araB*)567 deletion rendering it AraBAD(−), while the *ΔmetB* strain is AraBAD(+). This allows for visual discrimination of strains on tetrazolium arabinose (TA) agar (10 g/l tryptone, 1 g/l yeast extract, 5 g/l NaCl, 16 g/l agar, 10 g/l L-arabinose, 0.005% tetrazolium chloride), where the *ΔmetJ* strain forms red colonies and the *ΔmetB* strain forms white colonies^61^. Complete genomes of each strain are available for download at the Behringer Lab Github: [https://github.com/BehringerLab/Ecotype_Coexistence]

### Growth Curves

To conduct analysis of growth curves, strains were cultured overnight in 2 mL of modified M9 minimal medium (mM9) (119.39 mM disodium phosphate (anhydrous), 55.11 mM monopotassium phosphate, 21.39 mM sodium chloride, 46.74 mM ammonium chloride, 1 mM magnesium sulfate, 0.1 mM calcium chloride, and 20% w/v glucose). Overnight cultures were incubated at 37°C with shaking at 180 rpm in 16 × 100 mm borosilicate glass tubes. To assess the methionine auxotroph (*ΔmetB*) in monoculture, the mM9 media for overnight culture was supplemented with 5.9 mM L-methionine, matching the concentration of L-methionine in LB-Miller broth^62^. Cells from overnight culture were harvested by centrifugation of 1 mL of culture for 60 seconds at 10,000 rpm before washing twice with 1 mL fresh mM9 to remove residual metabolites and methionine. Washed overnight cultures were then diluted 1:10 dilution into fresh mM9 media, reflecting the subculture volume used in the 90-day co-culture experiment. For growth curve measurement, 100 uL aliquots were dispensed into wells of a 96-well cell culture plate (Greiner Cellstar; Product #: 655180) and absorbance at 600 nm (OD600) was recorded every 15 minutes for 20 hours using an Epoch 2 plate reader (BioTek) with double orbital shaking at 180 rpm and 37°C.

To generate conditioned media for verification experiments in Fig. 1B, a single colony was inoculated into 500 mL mM9 and incubated at 37°C with shaking at 180 rpm for 24 h in a 1 L Erlenmeyer flask. Cultures were then filtered through a 0.2 μm aPES vacuum filter unit (Fisherbrand; Product #: FB12566504) to remove cells. To assess growth curves in conditioned media, cultures were cultured overnight (18 h) shaking at 180 rpm at 37 °C in 2 mL of the filtered conditioned media supplemented with 0.2% glucose. These cultures were washed as previously described and then diluted 1:100 into fresh conditioned media. As before, 100 μL aliquots were transferred to a 96-well plate and growth curves were recorded with an Epoch 2 plate reader. Growth curve parameters (e.g. carrying capacity, growth rate) were calculated using the R package *growthcurver* (v.0.3.1)^63^.

### Culture conditions, co-culture experiment, and pairwise fitness assessment

Ancestral strains of the methionine auxotroph (*ΔmetB*) and methionine overproducer (*ΔmetJ*) were revived by streaking glycerol stocks onto LB-Miller agar plates (10 g/l tryptone, 5 g/l yeast extract, 10 g/l NaCl). For each mutant, 12 individual colonies were picked and inoculated into separate 16 × 100 mm glass culture tubes containing 10 mL of LB-Miller broth. Cultures were grown at 37°C with shaking at 180 rpm for 18 hrs. A glycerol stock was prepared for each of the 12 overnight cultures (1 mL culture + 200 μL 70% glycerol) to preserve the experimental starting populations.

To initiate the co-cultures, each overnight culture was centrifuged (1 mL, 10,000 rpm, 60 seconds), and the supernatant was discarded. Cell pellets were washed twice with 1 mL of fresh mM9 minimal medium to remove residual LB components, including amino acids and metabolic waste, ensuring standardized starting conditions. The final cell suspensions were adjusted to an OD600 of 0.4. Co-cultures were assembled by combining 100 μL of each washed *ΔmetB* and *ΔmetJ* strain (matched pairs from colonies 1–12) into fresh mM9 medium. Cultures were propagated in 16 × 100 mm glass tubes containing 10 mL of mM9 supplemented with 0.2% glucose. Tubes were incubated at 37°C with shaking at 180 rpm for the duration of the 90-day experiment. Subcultures were performed every 2 or 10 days by homogenizing the culture by vortexing and inoculating 1 mL into 9 mL of fresh medium. Two cell pellets and a glycerol stock were collected and flash-frozen every subculture for the first 30 days of co-culture, then every 10 days through day 90 for archival purposes. All strains are stored at −80°C and are available upon request. To monitor population dynamics, colony-forming units (CFUs) were measured each subculture by spot-plating 5 μL of serially diluted culture onto both tetrazolium arabinose (TA) agar and LB supplemented with 50 μg/mL kanamycin for populations 1, 2, and 3 in both subculture treatments. For populations 4–12, a glycerol stock generated every 10 days was completely de-thawed, homogenized, and 100 μL sampled before being re-frozen. Cells were enumerated by spot plating 6 μL (adjusted to 6 from 5 μL as the glycerol stock volume was 1200 μL) the serial diluted sample on tetrazolium agar. To facilitate statistical analysis and avoid zero values in the dataset, any timepoint at which an ecotype cell count was deemed uncountable was assigned a value of 0.1 at the lowest dilution factor where the same ecotype yielded a countable result in parallel populations.

To assess relative fitness of the methionine auxotroph (*ΔmetB*) and overproducer (*ΔmetJ*), we conducted competitive fitness experiments and calculated selection rate (r)^64,65^. Here, strains were grown overnight in 10 mL of mM9 medium at 37°C, shaking at 180 rpm in 16 × 100 mm glass culture tubes. Overnight cultures were washed twice with fresh mM9 as described above and normalized to OD600 = 0.4. Competitions were initiated by mixing 100 μL of *ΔmetB* with 150 μL of *ΔmetJ* per replicate. The unequal volumes were used to compensate for the higher observed mortality rate of *ΔmetJ* in mM9 medium, resulting in an approximately equal number of viable cells at the start of each competition. For competitions that required supplemented methionine, 5.9 mM L-methionine was added to the mM9 media before inoculation. CFUs were enumerated at days 0, 1, 2, 4, 7, and 10 by plating on TA agar. Selection rate (r) was determined as the difference of the natural log of the ratio of each competitor’s CFU counts at the final and initial time points:

$$r=\;\left[ln\left(\frac{{\text{StrainA}}_{\text{Final}}}{{\text{StrainA}}_{\text{Initial}}}\right)-l\;n\left(\frac{{\text{StrainB}}_{\text{Final}}}{{\text{StrainB}}_{\text{Initial}}}\right)\right]/\left(\#\text{of}\;\text{days}\right)$$

### Quantification of glucose and methionine

To quantify extracellular L-methionine from the overproducer, the *ΔmetJ* strain was grown overnight in 10 mL of mM9 minimal medium at 37°C, shaking at 180 rpm for 18 h in 16 × 100 mm glass culture tubes. For metabolite sampling, 1 mL of culture from each overnight (3 biological replicates) was subcultured directly—without washing—into 9 mL of fresh mM9 medium (+0.2% glucose) in a glass tube. Cultures were sampled longitudinally from the same tube at multiple timepoints throughout growth: 0, 2, 4, 6, 10, 14, and 18 hours. These timepoints were selected based on growth dynamics observed in glass tubes following a 1:10 dilution from 18-hour overnight cultures into fresh mM9 medium (Fig. 2B).

At each timepoint, 0.5 mL of culture was collected and centrifuged using Amicon Ultra 0.5 mL Ultracel 10K centrifugal filters (Product #: UFC501096) at 10,000 rpm for 5 minutes to remove cellular debris. The resulting filtrate was flash-frozen in liquid nitrogen and stored at −80°C for later analysis. Samples were submitted to the Vanderbilt Mass Spectrometry Research Center for LC-MS-based metabolite quantification. Amino acids were quantified as dansyl derivatives by reverse phase LC-MS using a Waters Acquity HPLC system interfaced with Thermo TSQ Quantum mass spectrometer. Selected ion monitoring (SIM) was performed using positive electrospray ionization^66^.

To quantify extracellular glucose as the overproducer grew, the *ΔmetJ* strain was grown overnight in 10 mL of mM9 minimal medium at 37°C, shaking at 180 rpm for 18 h in 16 × 100 mm glass culture tubes. For metabolite sampling, 1 mL of culture from each overnight (3 biological replicates) was subcultured directly—without washing—into 9 mL of fresh mM9 medium (+0.2% glucose) in a glass tube. Cultures were sampled longitudinally from the same tube at multiple timepoints throughout growth: 0, 2, 4, 6, 10, 14, and 18 hours. These timepoints were selected based on growth dynamics observed in glass tubes following a 1:10 dilution from 18-hour overnight cultures into fresh mM9 medium (Fig. 2B). At each timepoint, 0.5 mL of culture was collected and centrifuged using Amicon Ultra 0.5 mL Ultracel 10K centrifugal filters (Product #: UFC501096) at 10,000 rpm for 5 minutes to remove cellular debris. The resulting filtrate was flash-frozen in liquid nitrogen and stored at −80°C for later analysis. When ready to be analysed, samples were dethawed on ice until completely liquid. Using the Abcam Glucose Assay Kit (Product #: ab65333), glucose was quantified using a colorimetric reaction. Samples from hours 0 through 6 were tested undiluted and in three ten-fold dilutions, hours 10–18 were assessed directly with no dilutions. Reaction was measured using absorbance at 570 nm and concentrations back calculated using the resulting standard curve. All measurements were collected in duplicate, and the average value between the two readings was reported.

### Overproducer viability and auxotrophic growth experiments

Cultures to be measured for viability were inoculated with 100 μL of washed overproducer *ΔmetJ* cells from an overnight grown for 18 hours. For each media conditioning time (days 1–10), 6 replicate cultures were initiated from a single 18 hr overnight in 10 mL of mM9 media (+0.2% glucose). Cell density was assessed at 24 hours for every culture by enumerating CFUs, and was again enumerated at the endpoint for cultured aged from 1 to 10 days before viability staining. At each collection endpoint, each replicate culture’s cell viability was assessed by live/dead cell staining using the Invitrogen LIVE/DEAD BacLight Bacterial Viability Kit (Product #: L7012) and measuring the fluorescence emission ratio at 530/630 nm. The remaining 9 mL of culture in each of the 6 replicate cultures was then pooled and filtered through a 0.2 μm aPES vacuum filter unit (Fisherbrand; Product #: FB12566504) to remove cells and generate aged spent media.

To perform spent media growth curves with the auxotroph, the auxotroph was first grown overnight in 2 mL of mM9 media (+ 0.2% glucose, +5.9 mM L-methionine) for 18 hours. Next, 1 mL of culture was collected and the cell washed twice (as described previously) with fresh mM9 without methionine. Washed auxotrophic cells were then inoculated 1:100 into aged media and lightly vortexed to homogenize the mixture. 100 μL of the inoculant mixtures were then aliquoted into each well of a 96-well cell culture plate (Greiner Cellstar; Product #: 655180) and absorbance at 600 nm (OD600) was recorded every 15 minutes for 20 hours using an Epoch 2 plate reader (BioTek) with double orbital shaking at 180 rpm and 37°C (Note: wells on the perimeter of the culture plate were not inoculated as to avoid erroneous readings due to the limited growth in unsupplemented spent media). Growth rates were calculated by fitting a logistic curve to optical density data using the R package *growthcurver* (ver 0.3.1).

### Statistical Analyses

All statistical analysis and figure generation were conducted in R (version 4.4.2). Welch’s two-sided t-tests were performed using the *t.test()* function from the *stats* package (version 4.4.2). All t-tests were two-tailed. See figure legends for specific information on sample numbers and results.

### Mathematical model construction, data fitting, and assessment of chaotic behaviour

We model the auxotroph-overproducer interaction using a system of ordinary differential equations (ODEs) that describe consumer-resource dynamics. The core model consists of two species (an overproducer, X1, and an auxotroph, X2) and two resources (a primary nutrient, R1, and a metabolite, R2). The overproducer consumes R1 and produces R2 as a byproduct, while the auxotroph requires R2 for growth. Both species also compete for the primary resource, R1.

To simulate serial dilution experiments, we define a transfer function that is applied at fixed time intervals, *Δt*. This function instantaneously dilutes the culture by a factor and replenishes the primary resource, creating a discontinuous semi-flow. The state of the system immediately after each transfer defines a discrete-time map, which we analyse for its long-term behaviour.

Our fitting procedure is a multi-step, nested process designed to calibrate the model parameters to empirical data. We first fit a monoculture model of the overproducer to its growth curve data. We then fix these parameters and fit the remaining auxotroph-specific parameters to its growth data in co-culture, assuming no competition for the primary resource. Next, we introduce competition parameters and perform a global optimization on all parameters to best fit a full batch co-culture time series. Finally, to capture the non-convergent behaviour seen in serial passage data, we perform an additional calibration step. We sample from a bounded region around the previously fitted parameters and minimize the error in the relative species abundances across multiple dilutions.

To analyse the dynamics of the resulting discrete map, we compute its largest Lyapunov exponent to quantify sensitivity to initial conditions, a hallmark of chaos. We use established numerical methods to compute this exponent over many iterations of the map. Furthermore, to explicitly demonstrate the transition from stable to chaotic behaviour, we construct bifurcation diagrams using two different methods: (i) we linearly interpolate between a parameter set that produces stable, convergent dynamics and one that produces chaotic dynamics; (we use the dilution time, *Δt*, as the bifurcation parameter. For each value of the bifurcation parameter, we plot the long-term orbits of the discrete map, revealing a classic period-doubling cascade into chaos, which is corroborated by the Lyapunov exponent turning from negative to positive.

We also demonstrate that chaotic dynamics can emerge in a much simpler, generalized Lotka-Volterra (gLV) model subjected to a similar serial resampling process. In this framework, interactions are direct and pairwise, not mediated by resources. The resampling transfer function sets the initial condition for each new batch to be a fixed total biomass with proportions equal to the relative abundances from the end of the previous batch. For a two-species system, this reduces to a one-dimensional discrete map. We show that for certain sets of interaction coefficients, this simple map can also exhibit a period-doubling route to chaos, indicating that the phenomenon is not unique to complex consumer-resource models but can arise from simpler ecological interactions combined with periodic dilution. More details on all the steps in these procedures can be found in the Supplementary Information.

## Supplementary Material

Supplementary Files

This is a list of supplementary files associated with this preprint. Click to download.

- McLaughlinXBeardsleyetalNatureSupplementaryInformation.pdf
- MovieS1.mov

## Data Availability

All raw data and code needed to reproduce the figures in this study are available on Github. For empirical datasets and visualization code for all main figures, see https://github.com/BehringerLab/Ecotype_Coexistence. For data and code pertaining to the mathematical modelling and Supplementary Text, see https://github.com/tdbeardsley/chaos-in-microbial-models/. All other data are included in the manuscript and/or the Supplementary Materials.
